# Generating homogenous cortical preplate and deep-layer neurons using a combination of 2D and 3D differentiation cultures

**DOI:** 10.1038/s41598-020-62925-9

**Published:** 2020-04-14

**Authors:** Walaa F. Alsanie, Ola A. Bahri, Hamza H. Habeeballah, Majid Alhomrani, Mazen M. Almehmadi, Khalaf Alsharif, Ebaa M. Felemban, Yusuf S. Althobaiti, Atiah H. Almalki, Hashem O. Alsaab, Ahmed Gaber, Mohamed M. Hassan, Ana Maria Gregio Hardy, Qasim Alhadidi

**Affiliations:** 1 Department of Clinical Laboratories Science, The Faculty of Applied Medical Sciences, Taif University, Taif, Saudi Arabia; 2 The Deanship of Scientific Research, Taif University, Taif, Saudi Arabia; 3 Department of Nursing, The Faculty of Applied Medical Sciences, Taif University, Taif, Saudi Arabia; 4 Department of Pharmacology and Toxicology, College of Pharmacy, Taif University, Taif, Saudi Arabia; 5 Department of Pharmaceutical Chemistry, College of Pharmacy, Taif University, Taif, Saudi Arabia; 6 Department of Pharmacutics and Pharmaceutical technology, College of Pharmacy, Taif University, Taif, Saudi Arabia; 7 Addiction and Neuroscience Research Unit, Taif University, Taif, Saudi Arabia; 8 Department of Biology, Faculty of Science, Taif University, Taif, Saudi Arabia; 9 Department of Genetics, Faculty of Agriculture, Cairo University, Cairo, Egypt; 10 Department of Genetics, Faculty of Agriculture, Minufiya University, Shibin El Kom, Egypt; 11 Department of Physiology and Pharmacology, College of Medicine and Life Sciences, University of Toledo, Toledo, OH USA; 12 Department of Anesthesiology, Perioperative and Pain Medicine, Stanford Medical School, Stanford University, California, CA USA

**Keywords:** Cellular neuroscience, Development of the nervous system, Neurogenesis, Neuroscience

## Abstract

Embryonic stem cells (ESCs) can be used to derive different neural subtypes. Current differentiation protocols generate heterogeneous neural subtypes rather than a specific neuronal population. Here, we present a protocol to derive separate two-deep layer cortical neurons from mouse ESCs (mESCs). mESCs were differentiated into mature Tbr1 or Ctip2-positive neurons using a monolayer-based culture for neural induction and neurosphere-based culture for neural proliferation and expansion. The differentiation protocol relies on SMAD inhibition for neural induction and the use of FGF2 and EGF for proliferation and it is relatively short as mature neurons are generated between differentiation days 12–16. Compared with the monolayer-based differentiation method, mESCs can be directed to generate specific deep-layer cortical neurons rather than heterogeneous cortical neurons that are generated using the monolayer differentiation culture. The early analysis of progenitors using flow cytometry, immunocytochemistry, and qRT-PCR showed high neuralization efficiency. The immunocytochemistry and flow cytometry analyses on differentiation days 12 and 16 showed cultures enriched in Tbr1- and Ctip2-positive neurons, respectively. Conversely, the monolayer differentiation culture derived a mixture of Tbr1 and Ctip2 mature neurons. Our findings suggested that implementing a neurosphere-based culture enabled directing neural progenitors to adopt a specific cortical identity. The generated progenitors and neurons can be used for neural-development investigation, drug testing, disease modelling, and examining novel cellular replacement therapy strategies.

## Introduction

Embryonic stem cells (ESCs) are an invaluable research tool as they can differentiate into the three germ layers *in vitro*. The advancement in the differentiation protocols has enabled the derivation of different neural progenitors and mature neurons. These neurons can be used in disease modelling, drug testing, modelling development in a dish, and in establishing novel strategies for cellular replacement therapies. Although the interest has been shifted towards using human embryonic stem cells (hESCs), mouse embryonic stem cells (mESCs) still have a great potential in the field. Essentially, mESCs can be genetically manipulated where their differentiation is considerably faster than that of their human counterparts. Additionally, mESCs can be used in allogenic transplantation studies to avoid such issues associated with xenogeneic transplantation.

There are various differentiation protocols to derive either region-specific or heterogeneous neurons from mESCs^1,2^. These protocols rely either on the use of feeder-layer, monolayer cultures or the formation of embryoid bodies (EBs)^3–7^. The protocols implementing the co-culture method are affected by feeder patch-to-patch variability and the signals in these cultures are undefined^8,9^. Conversely, the differentiation of mESCs through the formation of embryoid bodies results in significantly heterogeneous cultures that contain cells from the three different germ layers^7,10,11^. However, we recently demonstrated an optimised differentiation protocol, which can be utilised to generate region-specific neurons from mESCs using monolayer differentiation cultures^1^. The cortical neural identity has been shown to be the default differentiation pathway in the absence of pattering factors *in vitro*^12^. By combining SMADs inhibitor and growth factors that stimulate proliferation in neural progenitors, we showed that naive mESCs adopted the cortical identity in monolayer-based cultures^1^.

Cortical developmental studies have identified various growth factors and numerous neural populations that structure this unique brain region. These developmental reports have paved the way for many studies that are trying to replicate these processes *in vitro*. Indeed, several protocols have been designed to generate cortical neurons from both hESCs and mESCs^12–15^. Although these protocols generate homogenous cortical neurons, there are no protocols yet to generate a layer specific neuronal population.

In this study, we compared monolayer and neurosphere-based differentiation cultures from naïve mESCs. Additionally, we optimised a novel differentiation protocol to generate two homogenous cortical populations separately. The differentiation protocol uses monolayer cultures for neural induction followed by the generation of neurosphere cultures used for proliferation and patterning. Subsequently, the mature neurons were generated in monolayer cultures after plating the differentiated neurospheres supplemented with maturation growth factors. Our protocol will facilitate the study of a specific deep cortical layer development. The generated neurons can be also used for other purposes such as disease modelling, drug testing, and cellular replacement therapies.

## Methods

### Embryonic stem cell line, cell culture maintenance, and pattering phase

The ES-E14TG2a mESC line (ATCC, Manassas, VA, USA) was used throughout this study. Cells were maintained in 2i media in a feeder-free culture supplemented with the GSK3 inhibitor CHIR9901 and MEK inhibitor PD0325901, respectively (see the supplementary information for full medium composition). When mESCs were 80% confluent, they were passaged and seeded into 0.2% gelatinised 48-well plates at a density of 5000 cells/well in basic LIF media (FBS media) (see the supplementary information for media composition). The undifferentiated mESCs were routinely tested for mycoplasma infection and checked for karyotypes abnormality.

In the following day of seeding cells (considered day 0), the media was changed to serum replacement-based media (SRM) containing LDN193189. Throughout the patterning phase (from days 1–5), cells were subjected to descending and ascending gradients of SRM and N2 media, being exposed to a number of growth factors as tabulated in Supplementary Table 1.

### Two-dimensional (2D) monolayer-based protocol

**Figure 1:**
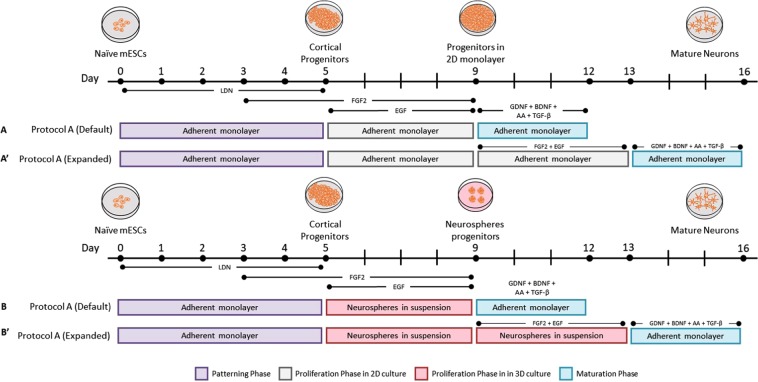
Schematic overview depicting the experimental design of the two neural induction protocols, highlighting the morphogens and small molecules employed in the current study. (**A**,**A′**) In the first protocol, mESCs were first patterned for 5 days, proliferated for 4 days -or (**A′**) passaged to be cultured for another 4 days, and lastly subjected to maturation for 3 days all of which were conducted in a 2-dimensional microenvironment. (**B,B′**) The second protocol was carried out in a similar fashion as the 2D protocol except that mESCs were cultured in a 3-dimensional microenvironment during the proliferation phase allowing them to grow in sphere-like structures (neurospheres).

After the pattering phase, the proliferation phase took place starting on day 5 and running for 4 (Fig. 1A,B) or 8 days (Fig. 1A′,B′). In this phase, the patterned mESCs [the so-believed to be cortical progenitor cells (CPCs) at this point] were detached with Accutase and seeded into the gelatinised wells that allowed them to proliferate in a 2D fashion. Cells were then cultured in a mixture of SRM/N2 media supplemented with FGF2 and EGF and changed every 2nd day (see the supplementary information for media composition, ratio, and concentrations).

Subsequently, cells were subjected to maturation for 3 days by culturing them on gelatinised wells in N2B27 media and exposing them to a panel of growth factors including glial cell-derived neurotrophic factor (GDNF), brain-derived neurotrophic factor (BDNF), ascorbic acid (AA), and transforming growth factor beta (TGF-β) (see Supplementary Table 1 for concentrations) (Fig. 1A,A′).

### Three dimensional (3D) neurosphere-based neural induction protocol

Similarly, the proliferation phase, herein, ran for two different periods. The default protocol ran for 4 days (Fig. 1B), whereas cells in the extended protocol were passaged for another 4 days of proliferation (Fig. 1B′). After collection of cells from the patterning phase using Accutase they were cultured in a non-treated culture dish in SRM/N2 media containing FGF2 and EGF to create neurospheres in a 3D fashion and the media was changed every 2nd day (see the supplementary information for media composition, ratio, and concentrations).

Afterwards, the derived neurospheres were plated in gelatinised 48-well plates. The neurospheres were cultured in an adherent culture for 3 days in N2B27 media being exposed to a number of growth factors similar to the monolayer-based neural induction protocol (Fig. 1B,B′).

### Flow cytometry analysis

The BD transcription factors staining kit (BD) was used to perform the intracellular staining. The staining was performed as previously described^1^. In brief, cells (1,000,000/tube) were incubated with BD Horizon fixable viability dye 450 (1:1000) diluted in phosphate-buffered saline (PBS) (at 4 °C, for 25 minutes). Cells were washed once with PBS, resuspended in fixation buffer (at 4 °C, for 45 minutes) and then washed three times with ice-cold permeabilization/washing buffer. Subsequently, they were incubated with the primary antibodies diluted in permeabilization/washing buffer (see Supplementary Table 2) overnight at 4 °C. After overnight incubation, the cells were washed three times in permeabilization/washing buffer and then incubated in secondary antibody (anti mouse-APC, 1:500, Santa Cruz, Heidelberg, Germany) for 45 minutes. After three washes in permeabilization/washing buffer, cells were resuspended in ice-cold 500 ul running flow buffer. The running flow buffer consisted of 1% bovine serum albumin (Sigma-Aldrich, St. Louis, MO, USA) and 0.5 mM EDTA (Sigma-Aldrich) diluted in PBS. The analysis of flow cytometry was performed using the BD Canto analyzer (BD), FACS DIVA (BD) and Flowlogic (Inivai technologies, Mentone, Victoria, Australia). Single cells were gated based on forward-side scatter profiles and dead cells were excluded using violet Horizon viability dye (BD) (see Supplementary Fig. 1). Undifferentiated mESCs were used as a negative control to set gates.

### Immunocytochemistry

Monolayer differentiation cultures (on days 5 and 14) and plated neurospheres were fixed and immunostained, using the previously described method^1^. Primary antibodies used are shown in Supplementary Table 3. Secondary antibodies, generated in donkey and conjugated to Alexa Flour 488, 555, and 649, were used at 1400 (Abcam, Cambridge, UK). Moreover, 4′, 6-diamidino-2-phenylindole (DAPI, Sigma-Aldrich) nuclear counterstain (1:1000) was used to visualise cells in differentiation culture. Images were captured using an inverted fluorescence microscope (Leica DMI8, Leica, Wetzlar, Germany).

### Quantitative real-time PCR

Total RNA was isolated using Trizol (Sigma-Aldrich). RNA was converted to cDNA and analysed using previously described methods quantitative real-time PCR (qRT-PCR)^16^. Primer sequences are described in Supplementary Table 4.

### Statistical analysis

Statistical analyses were carried out using GraphPad Prism (San Diego, CA, USA). Multiple comparisons were performed following Student’s t-test on datasets to determine significant differences between groups. Data are expressed as the mean ± standard error of mean (SEM). Differences are considered significant at a p value < 0.05. Significant differences are marked *, **, *** for p values < 0.05, <0.01, or <0.001, respectively.

## Results

### Confirmation of the naïve mESCs differentiation into neural cortical cells

**Figure 2:**
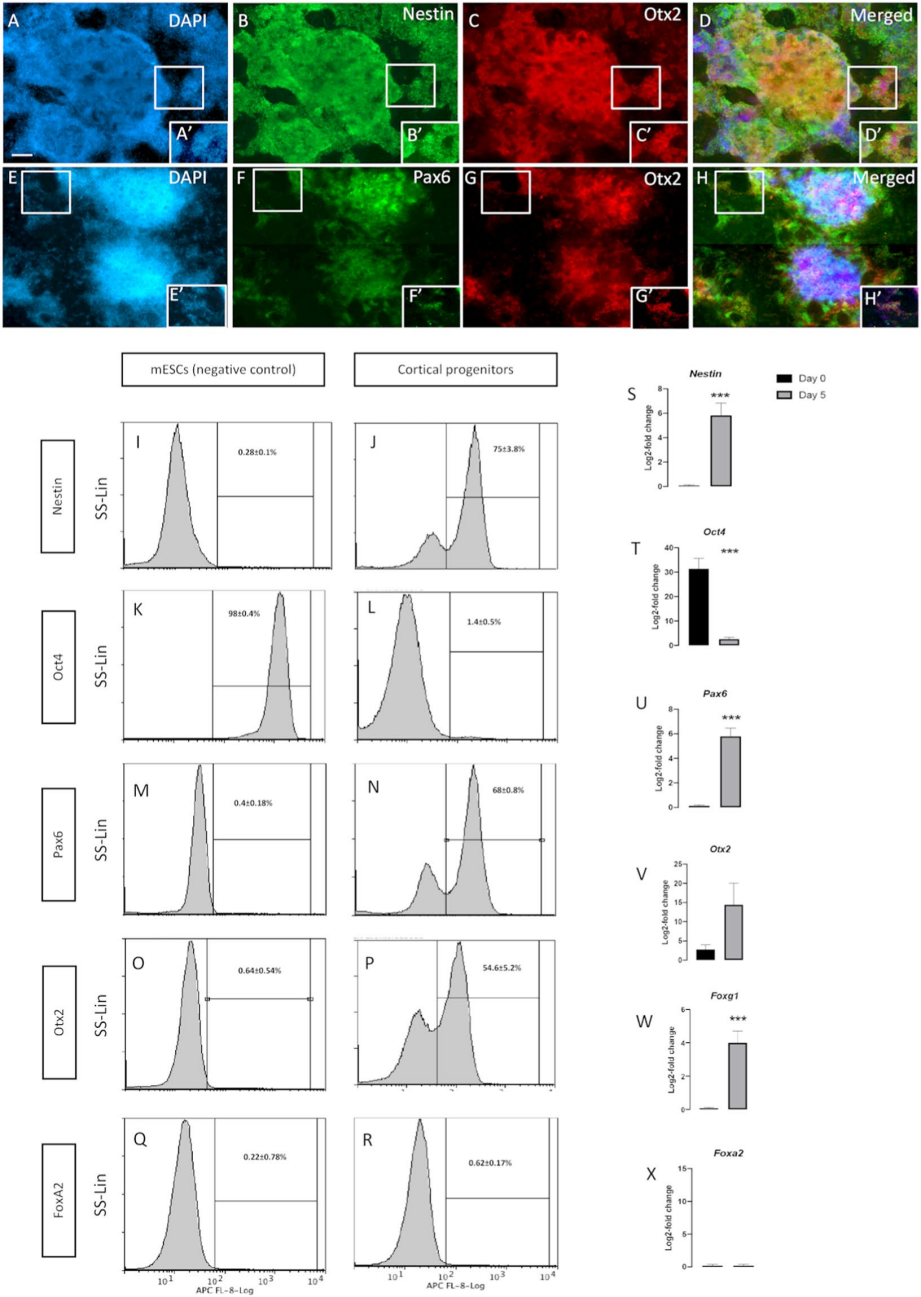
The naïve mESCs differentiated into cortical progenitors in 5 days in monolayer culture. The tile and zoomed images show that the differentiated neural progenitors, labelled with (**A,A′**) DAPI, widely express the neural progenitor marker (**B,B′**) Nestin and the forebrain/midbrain marker (**C,C′**) Otx2. (**D,D′**) Merged images. The lower panel of the photomicrographs show that neural progenitors stained with (**E,E′**) DAPI, highly express the early cortical marker (**F,F′**) Pax6 and the forebrain/midbrain marker (**G,G′**) Otx2. (**H,H′**) Merged images. Flow cytometry comparative analysis between mESCs (control) and neural progenitors on day 5 depict (**I**,**J**) dramatic elevation in Nestin expression, (**K**,**L**) disappearance of Oct4, emergence of both (**M**,**N**) Otx2 and (**O**,**P**) Pax6 in cortical progenitors, all of which when compared to their negative counterparts. (**Q**,**R**) The null expression FoxA2 in both cortical progenitors and their negative matches. Log-2-fold change in the transcriptional expression of (**S**) *Nestin*, (**T**) *Oct4*, (**U**) *Otx2*, (**V**) *Pax6*, (**W**) *Foxg1*, and (**X**) *FoxA1* supporting the findings obtained from the flow cytometry analyses. Data represents mean ± SEM, n = 5, Students t-test ***p < 0.001. Scale bars = 100 um.

Naïve mESCs were maintained in 2i medium, which contained LIF and the two inhibitors of glycogen synthase kinase 3 (GSK3) and MAPK/ERK kinase (MEK). mESC cells were split and pre-cultured in serum-supplemented media on gelatinised wells when they reached 80% confluency. To induce the differentiation machinery that directs mESCs into CPCs were cultured in serum replacement media supplemented with LDN193189 for the first 5 days. FGF2 was added in this neuralisation step from days 3–5. By day 5, the majority of mESC cells showed a clear transit from the pluripotency phase towards a dorsal forebrain identity (Fig. 2). This was confirmed by using qRT-PCR, flow cytometry, and immunocytochemistry. The results showed efficient neuralization by day 5. This was evident by the vanished expression of octamer-binding transcription factor 4 (Oct4) (1.4 ± 0.5%) and the robust expression of the neural progenitor marker of Nestin (Fig. 2I–L). The majority of the derived progenitors (75 ± 3.8%) expressed Nestin (Fig. 2B,L). This was also confirmed by demonstrating significant increase in the gene expression of *Nestin* along with significant drop in *Oct4* expression in these progenitors (Fig. 2S,T). Furthermore, these progenitors were confirmed to adopt the cortical lineage as evident by their expression for the dorsal-forebrain marker of paired box protein (Pax6) (68 ± 0.8%), forebrain-midbrain marker orthodenticle homeobox 2 (Otx2) (54.6 ± 5.2%), and the absence of the off-target ventral floor-plate progenitors marked by FoxA2 (0.62 ± 0.17%) (Fig. 2E–H,M–R). Importantly, the majority of Nestin-positive progenitors co-expressed Otx2 and the high colocalization of Pax6 and Otx2 ensured the dorsal cortical identity (Fig. 2A–H). In agreement with the immuno-labelling and flow cytometry analysis, qRT-PCR demonstrated a significant expression of *Pax6* and the absence of *FoxA2* expression (Fig. 2V,X). Although *Otx2* expression was not significant, the expression was higher in the progenitors compared with the undifferentiated mESCs (Fig. 2U). This could be due to the fact that *Otx2* is expressed in a subset of mESCs^17^. *Foxg1*, a gene that is known to be expressed in early cortical development^18^, was significantly expressed in the derived progenitors (Fig. 2W). These results suggest that most differentiated cells adopted the identity of early cortical progenitors.

### Monolayer and neurosphere-derived neurons from naïve mESC cells

**Figure 3:**
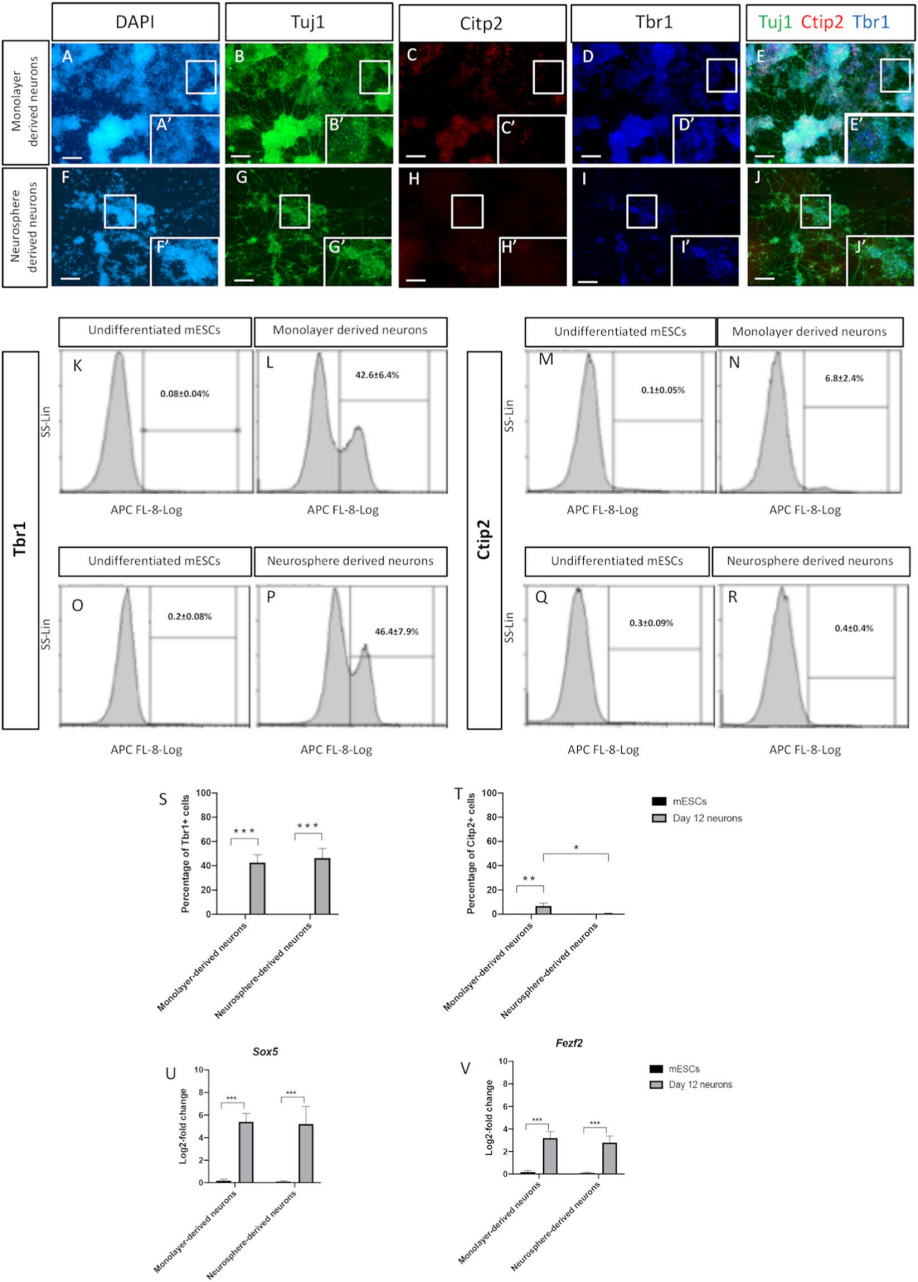
A panel of representative immunostaining images of 12 day old mature neurons derived from (**upper**) 2D monolayer and (**lower**) 3D neurosphere cultures. Monolayer-derived neurons labelled with (**A,A′**) DAPI, express (**B,B′**) the mature neuronal cytoskeletal protein Tuj1 and both of the mature cortical markers (**C,C′**) Ctip2 and (**D,D′**) Tbr1. (**E,E′**) Merged images. Neurosphere-derived neurons labelled with (**F,F′**) DAPI, showing their positivity for (**G,G′**) Tuj1 and (**I,I′**) Tbr1 but not (**H,H′**) Ctip2. (**J,J′**) Merged images. Flow cytometry comparative analysis of (**K–N**) Tbr1 and (**O–R**) Ctip2 expressions between monolayer- and neurosphere-derived neurons with their corresponding undifferentiated counterparts. A comparative analysis of the positively stained monolayer- and neurosphere-derived neurons for (**S**) Tbr1 and (**T**) Ctip2 compared to their undifferentiated counterparts. Log-2-fold change in the transcriptional expression of (**U**) *Sox5* and (**V**) *Fezf2* supporting the deep layer cortical identity. Data represents mean ± SEM, n = 5, Students t-test *p < 0.05, **p < 0.01, ***p < 0.001. Scale bars = 100 um.

Cortical progenitors were allowed to proliferate for 4 days while exposed to EGF and FGF2 growth factors in either adherent or suspension cultures. By day 9, the derived cortical progenitors formed a two-dimensional (2D) monolayer cell sheet or three-dimensional (3D) spheres-like structures. The neurospheres from day 9 were then transferred to gelatinised wells and supplemented with maturation media for another 3 days to undergo maturation. Similarly, the cortical progenitors in the 2D cultures, on day 9 of differentiation, were switched into maturation media for 3 days. On day 12, the derived neurons from both cultures (2D and 3D) were examined by flow cytometry and immunocytochemistry for their maturation (Fig. 3). Neurons derived from both 2D and 3D cultures (neurospheres) robustly expressed the neural cytoskeletal protein Tuj1, confirming their well differentiation and maturation (Fig. 3B,G). The monolayer-derived neurones expressed both the mature cortical preplate marker T-box brain 1 (Tbr1) and deep layer marker coup-TFI interacting protein 2 (Ctip2) (Fig. 3A–E). The majority of these neurons expressed Tbr1 (42.6 ± 6.4%), while the number of mature neurons expressing Ctip2 (6.8 ± 2.4%) was significantly lower than that of those expressing Tbr1 (Fig. 3K,L,O,P). In contrast, neurosphere derived neurons only showed expression for Tbr1 (Fig. 3F–J). The neurosphere-based differentiation culture showed similar levels of Tbr1-positive neurons (46.4 ± 7.9%), while there were almost no Ctip2 positive neurons (0.4 ± 0.4%) (Fig. 3M,N,Q,R). Although there was no significance difference in the number of Tbr1-positive neurons generated from both differentiation cultures at this time point, the subtle elevation in Ctip2 expression shown in the 2D derived neurons was significant (p < 0.5) compared to its elusive value presented by the neurosphere derived neurons (Fig. 3S,T). *Sox5* and *Fezf2*, which are genes expressed in the early development of deep cortical layers, were significantly expressed in neurons derived from both differentiation cultures (Fig. 3U,V).

### Prolonged differentiation of cortical progenitors

**Figure 4:**
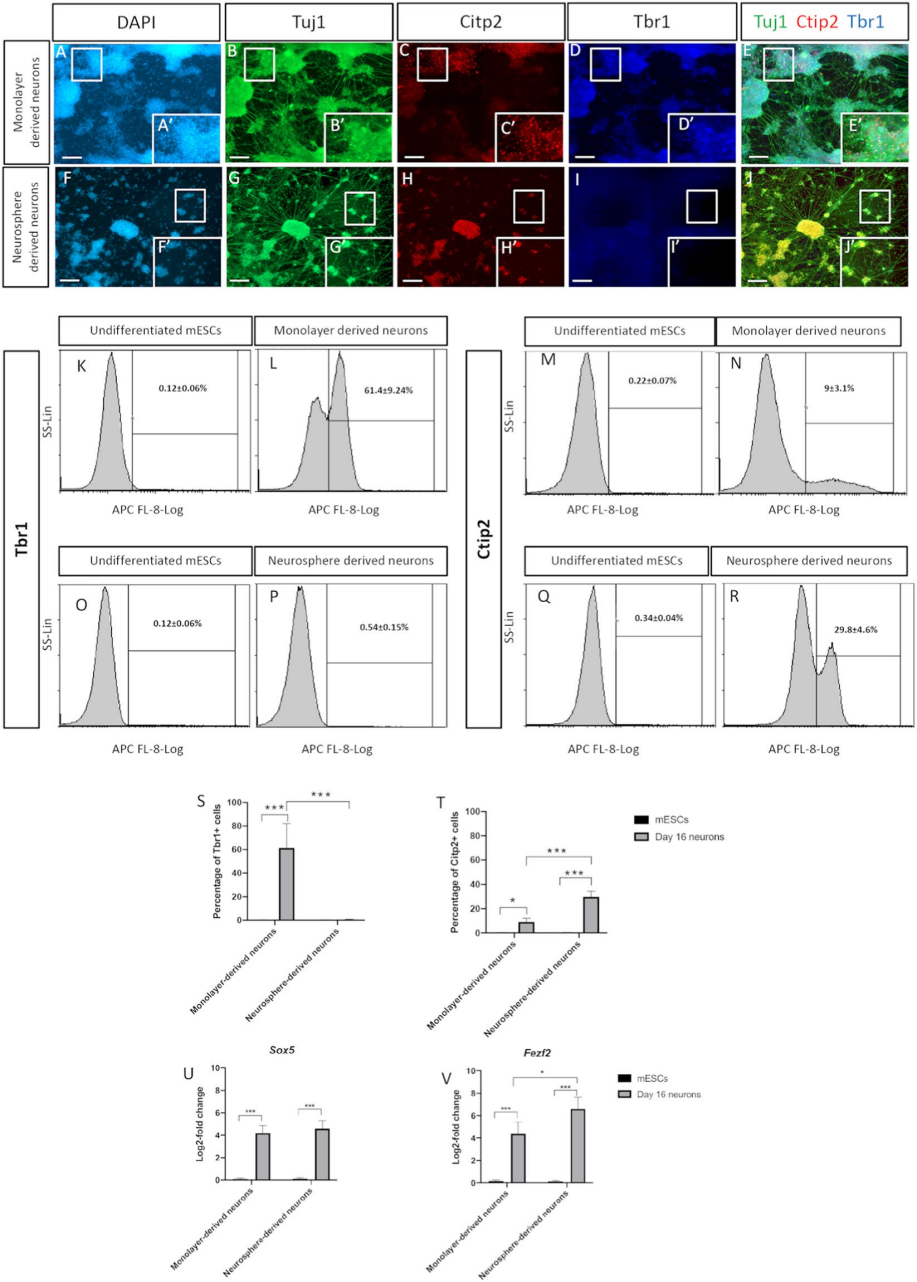
A panel of representative immunostaining images of 16 day old mature neurons derived from (**upper**) 2D monolayer and (**lower**) 3D neurosphere cultures. Monolayer-derived neurons labelled with (**A,A′**) DAPI, confirming their maturation by expressing (**B,B′**) Tuj1 and further confirming their cortical linage being positive for (**D,D′**) Tbr1 and with modest expression for (**C,C′**) Ctip2. (**E,E′**) Merged images. Neurosphere-derived neurons labelled with (**F,F′**) DAPI, showing their positivity for (**G,G′**) Tuj1 and (**H,H′**) Ctip2 but not (**I,I′**) Tbr1. (**J,J′**) Merged images. Flow cytometry comparative analysis of (**K**–**N**) Tbr1 and (**O**–**R**) Ctip2 expressions between monolayer- and neurosphere-derived neurons with their corresponding undifferentiated counterparts. A comparative analysis of the positively stained monolayer- and neurosphere-derived neurons for (**S**) Tbr1 and (**T**) Ctip2 comparing to their undifferentiated counterparts. Log-2-fold change in the transcriptional expression of **(U)** *Sox5* and **(V)** *Fezf2* supporting the deep layer cortical identity. Data represents mean ± SEM, n = 5, Students t-test *p < 0.05, ***p < 0.001. Scale bars = 100 um.

By extending the proliferation phase using FGF2 and EGF for another 4 days (8 days proliferation phase in total), the derived neurons generated from monolayer and neurosphere cultures yielded robust expression of the neural maturation marker Tuj1 after 3 days in maturation media (at day 16). There was no difference noted in Tuj1 expression compared to the corresponding expression in neurons generated on day 12. Monolayer-derived neurons on day 16 were similar to the neurons generated on day 12 regarding the expression pattern of Tbr1 and Ctip2 (Fig. 4A–E). Most neurons generated from the monolayer-based differentiation culture were Tbr1-positive (61.4 ± 9.24%), while a small population was positive for Ctip2 (9 ± 3.1%) (Fig. 4K,L,O,P). However, this was not the case in neurons derived from neurosphere-based differentiation cultures. Interestingly, there was almost no expression detected for Tbr1 in the neurons generated from neurospheres on day 16 while Ctip2-positive neurons existed widely in culture (Fig. 4F–J). In both cultures, the intermediate progenitor marker, Tbr2, was broadly expressed (see Supplementary Fig. 2). Previous studies have shown that Tbr2-positive progenitors are involved in the development of all cortical layers^19–22^. The quantitative analysis using flow cytometry confirmed the findings of immunocytochemistry showing that there were almost no Tbr1-positive neurons (0.54 ± 0.15%) in the differentiation culture derived using neurospheres, whereas the number of Ctip2-positive neurons (29.8 ± 4.6%) had increased considerably compared to the mature neurons generated on day 12 using the same differentiation culture style (Fig. 4M,N,Q,R). Although there was no significant difference between the neurons generated from both monolayer and neurosphere-differentiation cultures in terms of Tbr1 expression on day 12, the number of Tbr1-positive neurons was significantly higher in monolayer-derived than in neurosphere-generated neurons on day 16 (Fig. 4S). Furthermore, Ctip-2 positive neurons were significantly more frequent in the neurosphere-based than in the monolayer culture after the maturation phase on day 16 (Fig. 4T). Ctip2 is not exclusively expressed in the neurons of layer 5 of the cortex, as it is also expressed in the medium spiny neurons, which are generated from the lateral ganglionic eminence^23^. In both differentiation cultures, the derived Ctip2-positive neurons were negative for the medium spiny neuronal marker Darpp32 to confirm the deep cortical layer identity (see Supplementary Fig. 3). In addition, the two deep cortical layer-related genes, *Sox5* and *Fezf2*, were significantly expressed in the derived neurons from both differentiation cultures (Fig. 4U,V). Interestingly, the expression of *Fezf2* was higher in the neurons derived from the neurosphere-based culture than in those derived from the monolayer based-culture on day 16 (Fig. 4V).

## Discussion

The cerebral cortex is one of the most complex and well-organised structures in mammalian brain consisting of various neural cell types that are arranged in six different layers each of which comprises distinct cell populations^24^. In an orchestrated temporal order, cortical progenitor give rise to mature neurons following the so-called process “neuronal migration” filling and building up the six layers where each cell population carries a distinguishable morphology, maker proteins, and special pattern of gene expressions^12,25,26^. However, the critical mechanisms implicated in precisely orienting such diversity remain poorly understood. Therefore, a great number of studies and protocols have been reported in the literature through the past 2 decades attempting to untangle the complexity of the precise mechanism of corticogenesis by studying it *in vitro* using ESCs. Hence, various approaches were developed, e.g., to investigate efficient methods for differentiating ESCs into distinct regional neural populations, examining the ideal cocktail of morphogens and growth factors or manipulating the microenvironment of differentiation culture^1,13,27^. Interestingly, serious attempts have been made to study the employment and the differences between 2D or 3D cultures^28,29^. Zhou *et al*. demonstrated that culturing induced pluripotent stem cells (iPSCs) in 3D conditions resulted in higher expression of both neural progenitor (Pax6 and NESTIN) and astrocyte (GFAP and AQP4) markers as well as yielding a more homogenous cell population compared to those iPSCs cultured in 2D fashion^30^. Recently, András Dinnyés’s team reported that iPSCs neural induction in 3D produced a higher number of neural progenitor cells and neurons with longer neurites^29^. Nevertheless, many obstacles are believed to be involved in yielding regional-specific populations contaminated with undesirable cell types; the lack of direct comparisons between 2D and 3D environments, and other large variations in outcomes need to be further addressed.

The approach of using mESCs has long been approved as an advantageous tool facilitating the study of neural development and enabling prolific research in such fields including neurobiology, disease modelling, and drug testing. Nowadays, there are various techniques and protocols to guide mESCs to generate different neural cell types. For example, mESCs can be differentiated into cortical neural cells by simply culturing them in morphogen-free medium after blocking the cellular response of the hedgehog signalling using the steroidal alkaloid cyclopamine^12^. Recently, a study by Parish’s team demonstrated the feasibility of mESCs to derive cortical neural progenitors and mature neurons that were positive for Tbr1 only^1^. However, there was no other mature cortical marker detected by the end of the protocol. Therefore, we investigated the possibility of utilizing a neurosphere-based culture to derive distinct mature deep layer populations.

Neural induction can be highly affected by the microenvironmental niche and the fate of ESC types can be fundamentally altered when being cultured in different culture environments. Here, we relied on the neural induction protocol that we previously described^1^. Five days post patterning of naïve mESCs, the generated progenitors efficiently downregulated the pluripotency marker Oct4. Additionally, those differentiated progenitors confirmed their neural identity by expressing the early neural marker Nestin. The adoption of early cortical identity was confirmed by showing the expression of Otx2 and Pax6 both at the gene and protein level. Both Pax6 and Otx2 are vital transcription factors for early corticogenesis^31–34^. Importantly, the ventral brain marker FoxA2 was not detected by day 5 of neural induction showing that no off-target existed in our differentiation culture. In our previous study, we showed that using naïve mESCs cultured in 2i instead of serum-based media decreased the contamination of differentiation culture with off-target progenitors^1^. This would explain the lack of necessity to add sonic hedgehog antagonists, such as cyclopamine, to cortical differentiation culture compared to other differentiation protocols^12^.

Using the progenitors generated on day 5 after neural induction, we investigated the direct differences between differentiating these progenitors into mature cortical neurons in 2D versus 3D environments. Further, we sought to assess which protocol would yield a more homogenous cell population.

The first protocol (2D) utilised the same gelatine-based microenvironment for proliferating the derived cortical progenitors and for their maturation. Meanwhile, the second protocol (3D) allowed the generated cortical progenitors to proliferate in a suspension microenvironment offering them a chance to form 3D sphere-like structure (neurospheres). However, these neurospheres were then plated for maturation in a 2D gelatine-based microenvironment. Additionally, each protocol had a prolonged version where the proliferation phase for cortical progenitors was extended for another 4 days each of which followed the corresponding microenvironment culture type.

To examine the microenvironment influence on the differentiation of cortical progenitors, we followed two different methods expanding them for 4 days in either 2D monolayer or 3D suspension fashion. It was previously reported that environmental cues may both influence the fate of specific cell types and enhance the overall outcomes^29^. In neuroscience research, it was reportedly demonstrated that 3D surroundings improve the differentiation of neural progenitor cells into neurons where studies showed a higher output of neural progenitors and mature neuron markers recorded from 3D differentiation cultures^29,35^. Rather than forcing them to expand in an artificial 2D culture, we believe that allowing the cortical progenitors to differentiate in an environment that mimics the 3D structure of the brain is actually playing a role in directing the identity of generated mature neurons.

Herein, we showed that the neural progenitors were treated for 4 days with Fibroblast growth factor 2 (FGF2) and epidermal growth factor (EGF) during the proliferation phase after neural induction. FGF-2 and EGF were shown to support the proliferation of neural progenitors^36,37^. After the proliferation phase, the cortical progenitors from both monolayer and neurosphere-based cultures were switched into the maturation phase for 3 days. The monolayer and neurosphere-based cultures generated high numbers of Tbr1-positive neurons. However, there was a slight emergence of Ctip2-positive neurons in the monolayer cultures highlighting some grade of heterogeneity in the monolayer 2D culture comparing to almost 0% in neurons derived from neurosphere 3D cultures. In the mammalian cortex, Tbr1-neurons represent the mature neuronal population in the preplate and layer 6 of the cortex that first appear in development^38,39^. Tbr1-positive neurons are critical for the migration of the other cortical neurons that appear later in development and structure of other layers of the cortex^38^. Moreover, Ctip2-positive neurons represent the neurons that reside in layer 5 of the cortex^40^. To confirm the deep cortical layer identity of the derived neurons, we also demonstrated that *Sox5* and *Fezf2* were upregulated on day 12 of differentiation. Sox5 and Fezf2 are two important transcription factors that are expressed during the early development of deep cortical layers and the preplate^40–42^. Previous work by Wang and colleagues showed that *Fezf2* overexpression enriched the forebrain neurons that were differentiated from mESCs^43^.

We then investigated whether prolonging the proliferation phase in the differentiation protocol would change the identity of the derived neurons. On day 16, the 2D monolayer cultures yielded a similar pattern with a modest elevation in the protein expression of Tbr1 and Ctip2. Surprisingly, the neurosphere-derived neurones on day 16 had an opposite pattern to that of those differentiated for 12 days. The differentiated cortical progenitors did not generate Tbr1-positive neurons, but were instead differentiated into neurons that were Ctip-2 positive. It was already known that different cortical neurons from the preplate and deep layers through the upper layers of the developing cortex are generated in a time-dependent sequential manner^44,45^. In addition, it was reported that the generation of deep layer Ctip2-positive cortical neurons requires the repression preplate neural marker Tbr1 expression *in vivo*^44,45^. Similarly, Gaspard and colleagues showed that different cortical neurons generated *in vitro* from mESCs are derived in a sequential manner mimicking the cortical development *in vivo*^27^.

The cortical neurons in our differentiation cultures on day 16 also expressed *Sox5* and *Fezf2*. There was no significant difference in *Sox5* expression between the neurons generated from the monolayer and those generated from the neurosphere cultures. However, *Fezf2* expression was significantly higher in the neurons derived from the neurosphere-based cultures than from the monolayer-based cultures. This could be due to the fact that Ctip2-positive neurons were significantly higher in the neurosphere-based differentiation culture than in the monolayer-based differentiation culture on day 16. Previous work has shown that the expression of Fezf2 is downregulated in layer six and the preplate in late embryonic and early postnatal cortical development^41,46^. Conversely, the expression of Fezf2 is maintained in layer 5 at these late developmental stages^41,47^.

Our findings agree with those of previous *in vivo* and *in vitro* reports showing the sequential development of the deep cortical layers. The prolonged proliferation phase in our neurosphere-based protocol has allowed the generation of the deep layer neurons that are positive for Ctip2 on the expense of the preplate Tbr1-positive neurons. This could not be achieved in the monolayer-based culture as they produced a mixture of the two populations; Tbr1- and Ctip2-positive neurons. These findings agree with those of the previous study by Gaspard *et al*., where they produced a mixture of cortical neurons using a 2D differentiation environment along with morphogen free culture except for using sonic hedgehog inhibitor. This could be attributed to the 3D environment supporting the proliferation of the progenitors and repression of the maturation process. However, the monolayer-based culture may induce spontaneous maturation despite the growth factors and media components. We believe that the 3D microenvironment has a positive influence on the differentiation of neurospheres in a fashion where they could mimic the sequential neuronal migration process of the mammalian brain development *in vivo* as shown by the sequential expression of Tbr1 and Ctip2,

Taken together, we believe that we are able to direct the cortical progenitors to adopt specific preplate (Tbr1) or deep layer (Ctipt2) using monolayer culture for neural induction and 3D neurosphere-based culture for proliferation. Our amended protocol could be used to study early deep layer corticogenesis *in vitro*. Further studies are warranted to investigate the ability of extending our protocol to generate upper layer cortical neurons and glia.

## Supplementary information

Supplementary Information.
