# Supplementary material for: Generating homogenous cortical preplate and deep-layer neurons using a combination of 2D and 3D differentiation cultures
Source: Sci Rep. 2020 Apr 14;10:6272. doi: 10.1038/s41598-020-62925-9 (PMC7156727; doi:10.1038/s41598-020-62925-9)
Supplement: Supplementary file 1 — Supplementary Information. [file 41598_2020_62925_MOESM1_ESM.docx]

**Generating homogenous cortical preplate and deep-layer neurons using a combination of 2D and 3D differentiation cultures**

Walaa F. Alsanie^1,7*^, Ola A. Bahri^2^, Hamza H. Habeeballah^2^, Majid Alhomrani^1^, Mazen M. Almehmadi^1^, Khalaf Alsharif^1^, Ebaa M. Felemban^3^, Yusuf S. Althobaiti^4,7^, Atiah Almalki^5,7^, Hashem Alsaab^6,7^, Ahmed Gaber^8,9^, Mohamed M. Hassan^8,10^, Ana Maria Gregio Hardy^11^, Qasim Alhadidi^12^

^1^Department of Clinical Laboratories Science, The Faculty of Applied Medical Sciences, Taif University, Taif, Saudi Arabia. ^2^The Deanship of Scientific Research, Taif University, Taif, Saudi Arabia. ^3^Department of Nursing, The Faculty of Applied Medical Sciences, Taif University, Taif, Saudi Arabia. ^4^Department of Pharmacology and Toxicology, College of Pharmacy, Taif University, Taif, Saudi Arabia. ^5^Department of Pharmaceutical Chemistry, College of Pharmacy, Taif University, Taif, Saudi Arabia. ^6^Department of Pharmacutics and Pharmaceutical technology, College of Pharmacy, Taif University, Taif, Saudi Arabia. ^7^Addiction and Neuroscience Research Unit, Taif University, Taif, Saudi Arabia. ^8^Department of Biology, Faculty of Science, Taif University, Taif, Saudi Arabia. ^9^Department of Genetics, Faculty of Agriculture, Cairo University, Egypt. ^10^Department of Genetics, Faculty of Agriculture, Minufiya University, Egypt. ^11^Department of Physiology and Pharmacology, College of Medicine and Life Sciences, University of Toledo, OH, USA. ^12^Department of Anesthesiology, Perioperative and Pain Medicine, Stanford Medical School, Stanford University, CA, USA

*Correspondent Author: Walaa F. Alsanie (w.alsanie@tu.edu.sa)

# Supplementary Methods and Tables

## Media composition:

### 2i media:

The 2i media consisted of knockout DMEM, 1X L-glutamine, 1X penicillin/streptomycin (P/S), 1X N2 supplement, 1X B27 minus vitamin A, 1X non-essential amino acids (NEAA) (all from Life Technologies), 110 uM beta-2- mercaptoethanol (Sigma), 2000IU/ml Leukemia inhibitory factor (LIF), 3 uM GSK3 inhibitor CHIR9901, 1 uM MEK inhibitor PD0325901 (all from Stem Cell Technologies).

### Basic LIF media (FBS media):

Basic LIF media consisted of knockout DMEM, 1X L-glutamine, 1X P/S, 1X NEAA (all from Life Technologies), 10% fetal bovine serum, 110 uM beta-2- mercaptoethanol (all from Sigma), 2000 IU/ml LIF (Stem cell technologies).

### Serum replacement-based media (SRM):

SRM consisted of DMEM/f12, 1X L-glutamine, 1X P/S, 1X NEAA, 1X insulin-transferrin-Selenium-sodium pyruvate (ITS-A), 15% knockout serum replacement (all from Life Technologies) and 110 uM beta-2-mercapthoethanol (Sigma).

### N2 media

N2 media consisted of DMEM/f12, 1X L-glutamine, 1X P/S, 1X NEAA, 1X insulin-transferrin-Selenium-sodium pyruvate (ITS-A), 1X N2 supplement (all from Life Technologies) and 110 uM beta-2-mercapthoethanol (Sigma).

### N2 B27 media

N2 B27 media consisted of 1:1 DMEM/f12 and neurobasal media, 1X N2 supplement, B27 supplement, 1X L-glutamine, 1X P/S, 1X NEAA, 1X ITS-A (all from Life Technologies) and 110 uM beta-2-mercapthoethanol (Sigma).

### Table 1. The details of the two protocols used in this study:

| **Protocol Type** | **Phase** | **Day No.** | **Media Composition** | **Added Supplements and Growth Factors** |
| --- | --- | --- | --- | --- |
| Maintenance | Maintenance | Pre- seeding | 2i media | GSK3 inhibitor CHIR9901 and MEK inhibitor PD0325901 (All from Stem Cell Technologies). |
| Default Protocol | Patterning | 0 | 100% SRM | 200 nM LDN193189 (Stem cell technologies) |
|  |  | 1 | 75% SRM + 25% N2 | LDN193189 (Stem cell technologies) |
|  |  | 2 | 50% SRM + 50% N2 |  |
|  |  | 3 – 5 | 25% SRM + 75% N2 | 20 ng/ml FGF2 (Peprotech) and 200 nM LDN193189 (Stem cell technologies) |
|  | Proliferation | 5 – 9 | 25% SRM and N2 media | 20 ng/ml FGF2 and 10 ng/ml EGF |
|  | Maturation | 9 – 12 | N2 B27 media | 30 ng/ml GDNF (R&D), 30 ng/ml BDNF (R&D), 1 ng/ml TGF-B and ascorbic acid (Sigma-Aldrich) |
| Extended Protocol | Patterning | 0 – 5 | Same as the default protocol | Same as the default protocol |
|  | Extended Proliferation | 5 – 9  9 – 13 | Same as the default protocol  Passaged every 4 days | Same as the default protocol |
|  | Maturation | 13 – 16 | Same as the default protocol | Same as the default protocol |

| Antibody | Species | Brand | Dilution |
| --- | --- | --- | --- |
| Oct4 | Mouse | BD | 1:20 |
| Pax6 | Mouse | DSHB | 1:250 |
| FoxA2 | Mouse | Abcam | 1:4000 |
| Otx2 | Goat | R&D | 1:1000 |
| Nestin | Mouse | Abcam | 1:2000 |

Table 2: List of primary antibodies used in flow cytometry:

Table 3: List of primary antibodies used for immunocytochemistry:

| Antibody | Species | Brand | Dilution |
| --- | --- | --- | --- |
| Nestin | Mouse | Abcam | 1:400 |
| Pax6 | Mouse | DSHB | 1:50 |
| Otx2 | Goat | R&D | 1:400 |
| Tbr1 | Rabbit | Abcam | 1:500 |
| Ctip2 | Rat | Abcam | 1:500 |
| Tuj1 | Mouse | Promega | 1:1000 |
| Tbr2 | Rabbit | Abcam | 1:500 |
| Darpp32 | Rabbit | Millipore | 1:400 |

Table 4: Primers used for RT-PCR:

| Gene | Primer |
| --- | --- |
| *Oct4* | F: AGTGAATGGGCGGAGTTATG |
|  | R: ACTTGGACGGGAACTGACAC |
| *Nestin* | F: AGGCTGAGAACTCTCGCTTGC |
|  | R: GGTGCTGGTCCTCTGGTATCC |
| *Otx2* | F: GCTGGCTCAACTTCCTACT |
|  | R: TCCAAGCAGTCAGCATTGAAG |
| *Foxa2* | F: GAGCACCATTACGCCTTCAAC |
|  | R: AGGCCTTGAGGTCCATTTTGT |
| *Pax6* | F: AGTGAATGGGCGGAGTTATG |
|  | R: ACTTGGACGGGAACTGACAC |
| *Foxg1* | F: TGGCAACACTGCCCATTCA |
|  | R: GCATTTGCGCAACACAGGTTA |
| *Sox5* | F: CAGCATGCTTACTGACCCTG |
|  | R: TCTCCTCCTCTTCCACTTTC |
| *Fezf2* | F: CCCTTCGTCTGCGAGTTT |
|  | R: TGTCCTGGCTAGGTCCTTT |
| *Hprt1* | F: GTTTGCTGACCTGCTGGATT |
|  | R: TATGTCCCCCGTTGACTGAT |


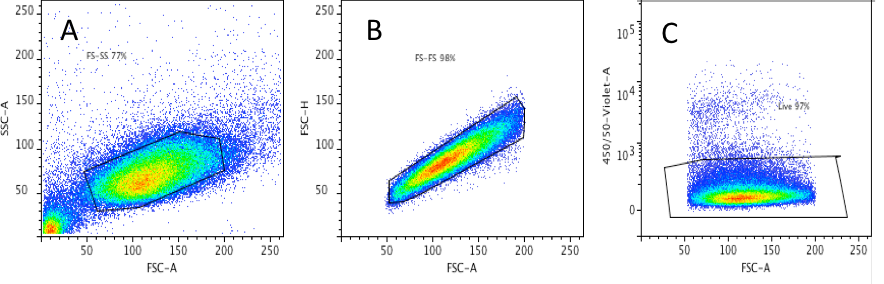


97%

98%

77%

**Supplementary figure 1.** Gating strategy for flow cytometry analysis. (A) The cells were gated using SSC-A and FSC-A to remove the clumps and cell debris. (B) The cells were gated again using FSC-H and FSC-A to assure that there are no clumps or debris in the analyzed population. (C) The live cells were gated to exclude the dead cells from the analysis.


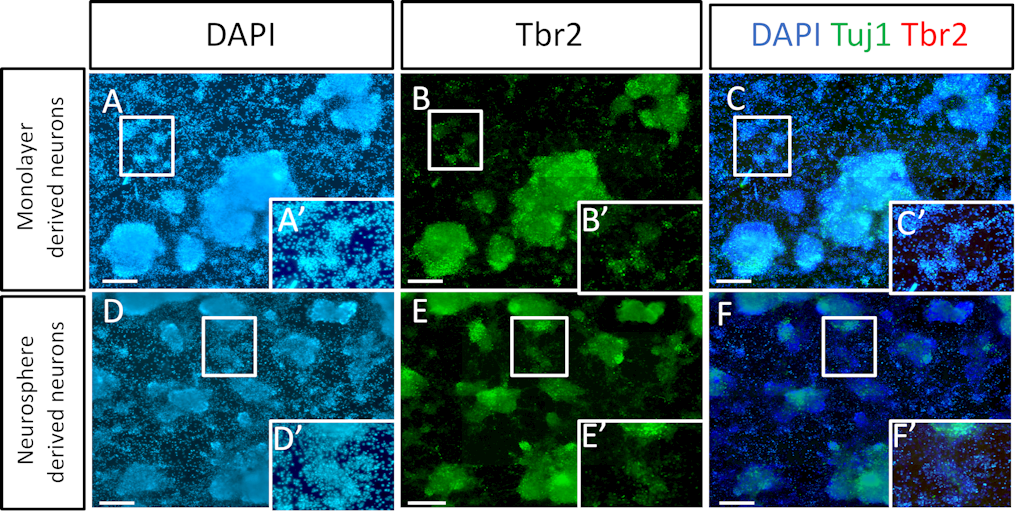


**Supplementary figure 2.** Tbr2-positive intermediate progenitors are expressed widely in monolayer- and neurosphere- derived neurons at day 16. The tile and zoomed images show that the differentiated neurons from monolayer and neurosphere based cultures, labelled with DAPI (**A, A’, D, D’**), widely express the intermediate progenitor marker Tbr2 (**B, B’, E, E’**). Merged images (**C, C’, F, F’**). Scale bars = 100 um.


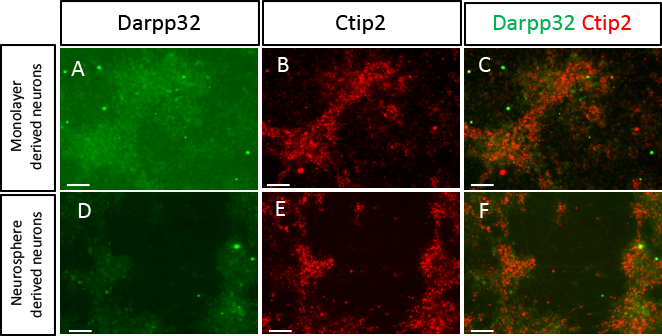


**Supplementary figure 3.** Ctip2-positive neurons are negative for Darpp32 in monolayer- and neurosphere-based cultures at day 16. The images show that the differentiated neurons, from monolayer and neurosphere based cultures, do not express Darpp32 (**A, D**), while Ctip2 is widely expressed in the neurons derived from both cultures (**B, E**). Merged images (**C, F**). Scale bars = 50 um.
